# Predictors of Distal Stent Graft-Induced New Entry after Frozen Elephant Trunk in Acute Type A Aortic Dissection

**DOI:** 10.1093/ejcts/ezaf264

**Published:** 2025-08-04

**Authors:** Junya Kitaura, Tomokuni Furukawa, Kazuki Maeda, Shingo Mochizuki, Kazunori Yamada

**Affiliations:** Department of Cardiovascular Surgery, Akane-Foundation Tsuchiya General Hospital, Hiroshima 730-0811, Japan; Department of Cardiovascular Surgery, Akane-Foundation Tsuchiya General Hospital, Hiroshima 730-0811, Japan; Department of Cardiovascular Surgery, Akane-Foundation Tsuchiya General Hospital, Hiroshima 730-0811, Japan; Department of Cardiovascular Surgery, Akane-Foundation Tsuchiya General Hospital, Hiroshima 730-0811, Japan; Department of Cardiovascular Surgery, Akane-Foundation Tsuchiya General Hospital, Hiroshima 730-0811, Japan

**Keywords:** distal stent graft-induced new entry, acute aortic dissection, frozen elephant trunk

## Abstract

**Objectives:**

Distal stent graft-induced new entry (d-SINE) is a significant complication of frozen elephant trunk (FET) for acute Stanford type A aortic dissection (ATAAD). This study aimed to identify the predictive factors for d-SINE occurrence.

**Methods:**

Patients who underwent FET procedures for ATAAD at our institution between January 2015 and December 2024 were retrospectively analysed. Patients were classified into d-SINE positive and d-SINE negative groups, and predictive factors were identified using Fine and Gray competing risk regression.

**Results:**

A total of 84 consecutive patients were included. The overall incidence of distal stent graft-induced new entries was 11.9% (10/84). Distal stent graft-induced new entry occurred at a mean of 102 days (standard deviation 69) postoperatively. A preoperative descending aortic diameter ≥33 mm was an independent predictive factor (subdistribution hazard ratio, 6.11; 95% confidence interval, 1.176-31.740; *P* = .031).

**Conclusions:**

A preoperative descending aortic diameter ≥33 mm was identified as a key predictor of d-SINE following FET for ATAAD. This finding may help identify high-risk patients who require more intensive imaging surveillance after surgery.

**Clinical Registration Number:**

E250127-4.

## Graphical Abstract

**Figure:**
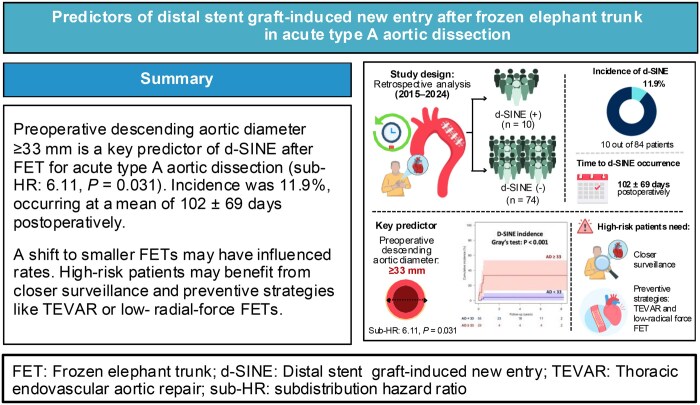

## INTRODUCTION

Although surgical outcomes for acute Stanford type A aortic dissection (ATAAD) have improved, the presence of complications or delays in treatment continues to render many cases high-risk.^1^ The frozen elephant trunk (FET) technique is increasingly adopted to promote false lumen (FL) thrombosis and reduce reintervention rates.^2–5^ However, it remains associated with a substantial risk of secondary aortic reintervention.^6–8^ A distal stent graft-induced new entry (d-SINE) is a serious complication that may necessitate emergency open surgery or thoracic endovascular aortic repair (TEVAR). Recent studies report a d-SINE incidence of 14.1%-27.9% following FET^9–11^; however, the true incidence in acute cases remains uncertain due to variations in follow-up protocols and imaging criteria. Although often asymptomatic and detected incidentally on CT, d-SINE can lead to re-dissection, rupture, and fatal outcomes.^12^ Predictive factors for d-SINE after TEVAR include stent graft (SG) oversizing, size mismatch, and procedural timing.^13–15^ Hiraoka et al identified a time of >48 h between the initial onset of ATAAD symptoms and the performance of surgical intervention as an independent risk factor.^10^ Additionally, FET for chronic dissection is associated with a higher incidence of d-SINE.^12^^,^^16^ However, the risk factors for d-SINE after FET in the setting of ATAAD remain unclear. This study aimed to identify preoperative clinical and anatomical predictors of d-SINE following FET repair for ATAAD, thereby supporting risk stratification, optimizing surgical strategies, and improving patient outcomes.

## METHODS

### Patient population

This retrospective study aimed to identify clinical and anatomical predictors of d-SINE following FET procedures for ATAAD. Data on aortic events (including aortic rupture, rapid aortic enlargement [>5 mm/year], new dissection, reintervention, or aortic-related death) and reintervention-related complications were also collected and reported descriptively.

Patients who underwent FET using J Graft Frozenix (Japan Lifeline Co Ltd, Tokyo, Japan) or J Graft Frozenix Partial ET (Japan Lifeline Co Ltd, Tokyo, Japan; hereafter referred to as PET) for ATAAD at our institution between January 2015 and December 2024 were retrospectively reviewed. Follow-up was completed in February 2025 for all patients included in the analysis.

A retrospective review of patient records was conducted to collect baseline characteristics, preoperative CT imaging findings, operative details, and postoperative outcomes. Patients were excluded if they underwent surgery more than 14 days after symptom onset, had a history of chronic aortic dissection, were unable to undergo follow-up CT due to postoperative mortality, or received an FET procedure using a device other than J Graft Frozenix or PET.

This retrospective clinical study was approved by the Institutional Review Board of Akane-Foundation Tsuchiya General Hospital (approval number: E250127-4, approval date: January 27, 2025), which granted a waiver of informed consent due to the retrospective nature of the study.

### Primary end-point

The primary end-point of this study was to identify clinical and anatomical predictors of d-SINE in patients undergoing FET for ATAAD.

### Device strategy and era-based treatment trends

J Graft FROZENIX and PET were used in this study.^17^^,^^18^ Both devices comprise a distal polyester graft reinforced with an elliptical nitinol stent and an unstented proximal segment. The PET includes an additional 20 mm unstented skirt distally, with the stented portion limited to 60 mm. Devices are available in 2-mm diameter increments (21-39 mm) and lengths of 60-150 mm (see **Figure S1**). According to the manufacturer, sizing should be approximately 110% of the TL diameter or 90% of the total aortic diameter. At our institution, FET size selection criteria differed between the Early and Late eras. In the Early era (before March 2023, *N* = 52), the selection was primarily based on 90% of the descending aortic outer diameter. In the Late era (April 2023 onward, *N* = 32), the criteria were adjusted to 80%-85% of the descending aortic outer diameter or 100%-105% of the maximum TL diameter to optimize outcomes. The increase in case volume during the late period was likely due to regional referral centralization and a wider adoption of the FET technique.

### FET procedure

All patients underwent median sternotomy. Arterial perfusion was established via the ascending aorta, femoral, or axillary artery. Antegrade-selective cerebral perfusion through the neck vessels preceded circulatory arrest. After rectal temperature reached 28°C, the aorta was transected between the left common carotid and subclavian arteries. The FET was deployed under blood-filled conditions using transoesophageal echocardiography. Distal anastomosis was performed, followed by total arch replacement with a 4-branched graft.

### Imaging analysis

**Figure 1.**
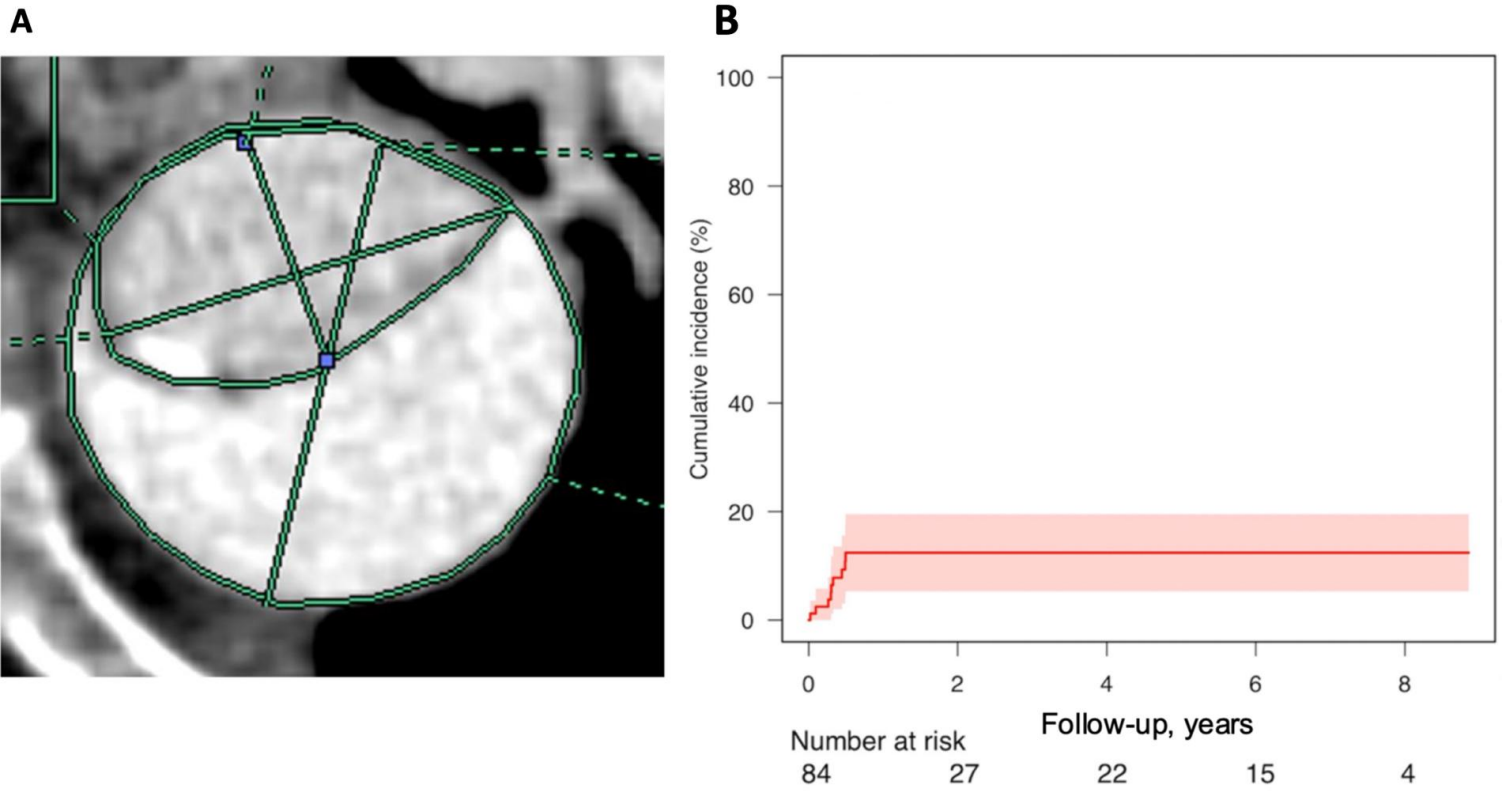
(A) Schematic of descending aortic and true lumen measurements, including diameter, circumference, and area. (B) Competing risk regression analysis for d-SINE occurrence, with death as a competing event. d-SINE, distal stent graft-induced new entry

Preoperative CT was used to evaluate the aortic morphology and FET size. Two cardiovascular surgeons retrospectively evaluated preoperative contrast-enhanced CT scans, resolving discrepancies by consensus. Measurements at the distal SG site included the outer diameter of the descending aorta at the intended distal landing zone of the FET. Additionally, the mean diameter of the descending aorta was calculated from orthogonal long- and short-axis measurements. At the same level, the maximum and minimum diameters of the TL were also measured. Circumference and cross-sectional area of both the descending aorta and TL were evaluated (**Figure 1A**). To evaluate the size mismatch between the SG and the native aorta, 2 key ratios were compared between the study groups: SG diameter relative to the mean descending aortic diameter (×100%) and SG diameter relative to the maximum TL diameter (×100%).

### Follow-up CT

The initial follow-up CT scan was performed 1-2 weeks postoperatively. Subsequent CT evaluations were conducted at 6 months, 1 year, and annually thereafter. In addition, 1-year postoperative changes in the aortic diameter at the distal SG site, as well as in SG diameter and cross-sectional area, were evaluated.

### Statistical analysis

The distribution of continuous variables was assessed for normality using the Shapiro-Wilk test. Normally distributed data are expressed as mean (standard deviation [SD]), and non-normally distributed data as median (interquartile range [IQR]). Categorical variables are presented as counts and percentages. To evaluate temporal changes in surgical strategy and outcomes, patients were stratified into 2 chronological cohorts, an Early era (January 2015 to March 2023) and a Late era (April 2023 to December 2024), based on the adoption of revised sizing protocols. Comparisons between these eras were performed using the chi-squared test or Fisher’s exact test for categorical variables, and the unpaired Student’s *t*-test or Mann-Whitney *U*-test for continuous variables, as appropriate. The cumulative incidence of d-SINE was analysed using competing risk analysis, considering death as a competing event, and group comparisons were conducted using Gray’s test. Additionally, Fine and Gray competing risk regressions were performed to estimate univariable subdistribution hazard ratios (sub-HRs), providing a competing risk-adjusted assessment of the impact of each covariate on d-SINE occurrence. To further explore the potential nonlinear association between preoperative descending aortic diameter and the risk of d-SINE, we incorporated a restricted cubic spline model with a limited number of knots into the Fine and Gray regression framework. Univariable and multivariable Fine and Gray competing risk regression analyses were conducted to identify predictive factors. Variables for multivariable analysis were selected based on both clinical relevance and univariable association with d-SINE (*P* < .10). Statistically significant variables were excluded if they showed collinearity, lacked clinical plausibility, or had insufficient events. Collinearity among covariates was assessed using variance inflation factors (VIFs), with a VIF >5 considered indicative of potential multicollinearity. Missing data were handled using multiple imputation techniques. Statistical significance was set at *P*-value <.05. All statistical analyses were conducted using EZR (version 1.68; Saitama Medical Centre, Jichi Medical University, Saitama, Japan), which is a graphical user interface for R (version 4.4.2; The R Foundation for Statistical Computing, Vienna, Austria). EZR is a modified version of R Commander, designed to incorporate statistical functions frequently used in biostatistics.^19^

## RESULTS

### Patient characteristics

Between January 2015 and December 2024, 84 consecutive patients who underwent FET for ATAAD using J Graft Frozenix or PET were enrolled from a total of 304 patients who underwent surgical treatment for ATAAD at our institution. The patient characteristics are summarized in **Table 1**. No baseline characteristics were found to be significantly associated with the occurrence of d-SINE.

**Table 1.** Baseline Patient Characteristics and Their Association with the Presence or Absence of Distal Stent Graft-Induced New Entry

Variable	*N* = 84	Univariable sub-HR (95% CI)	*P*-value
Age, years	61.7 (11.9)	1.052 (0.9943-1.112)	.079
Male	56 (66.7%)	4.83 (0.6294-37.07)	.13
BMI, kg/m^2^	24.5 (22.5-27.6)	0.927 (0.8366-1.027)	.15
BSA, m^2^	1.78 (1.60-1.90)	0.964 (0.891-1.043)	.36
Hypertension	78 (92.9%)	1.452 (0.414-5.098)	.54
Diabetes mellitus	9 (10.7%)	2.514 (0.548-11.53)	.24
Dyslipidemia	26 (31.0%)	1.452 (0.414-5.098)	.56
COPD	6 (7.1%)	Not available^a^	
CKD (eGFR < 60 mL/min/1.73 m^2^)	38 (45.2%)	0.821 (0.236-2.86)	.76
Haemodialysis	3 (3.6%)	Not available^a^	
History of stroke	6 (7.1%)	1.61 (0.188-13.79)	.66
Haemodynamic shock	7 (8.3%)	Not available^a^	
Site of primary entry		0.778 (0.417-1.451)	.43
Ascending	36 (42.9%)		
Arch	33 (39.3%)		
Proximal descending	8 (9.5%)		
Unknown	7 (8.3%)		
Condition of the false lumen		0.712 (0.277-1.83)	.48
Open	42 (50.0%)		
Partially thrombosed	34 (40.5%)		
Completely thrombosed	8 (9.5%)		
Follow-up period (years)	1.3 (0.74-5.0)	1.000 (0.999-1.001)	.64

a Due to no distal stent graft-induced new entry case detected in those with specific condition.

Abbreviations: BSA, body surface area; CI, confidence interval; CKD, chronic kidney disease; COPD, chronic obstructive pulumoary disease; d-SINE, distal stent graft-induced new entry; eGFR, estimated glomerular filtration rate; SD, standard deviation; sub-HR, subdistribution hazard ratio.

### Incidence of d-SINE

The overall incidence of d-SINE after FET was 11.9% (10/84 patients), and all cases occurred within the first 6 months postoperatively. According to the competing risk analysis, the cumulative incidence of d-SINE, with death considered as a competing event, was 13.4% at 1, 3, and 5 years, as shown in **Figure 1B**.

### Preoperative CT analysis

Descriptive values of preoperative descending aortic diameter, circumference, and TL morphology are summarized in **Table 2**. Among patients who subsequently developed d-SINE, the mean preoperative descending aortic diameter was 34.1 mm (SD 2.6), while in those who did not develop d-SINE, it was 31.0 mm (SD 3.1). These values are reported descriptively and were not formally compared between the groups, in order to minimize bias related to time-dependent outcomes. Instead, the predictive association of aortic diameter with d-SINE occurrence was formally assessed using Fine and Gray competing risk regression, which demonstrated a statistically significant association (sub-HR 1.258, 95% confidence interval (CI) 1.081-1.462, *P* = .003).

**Table 2.** Preoperative CT Parameters and Operative Data, and Their Association with d-SINE

Variable	*N* = 84	Univariable sub-HR (95% CI)	*P*-value
Size of the descending aorta			
Mean diameter (mm)	31.4 (3.2)	1.258 (1.081-1.462)	.003
Circumference (mm)	104.6 (10.8)	1.068 (1.026-1.112)	.001
Area (mm^2^)	791(715-932)	1.014 (1.001-1.006)	.002
Aortic diameter ≥33 (mm)	29 (34.5%)	8.629 (1.866-39.91)	.006
Size of the TL			
Maximum diameter (mm)	25 (22-27)	1.095 (0.981-1.222)	.11
Minimum diameter (mm)	15 (10-19)	1.05 (0.981-1.124)	.16
Circumference (mm)	68.5 (61-77)	1.032 (1.002-1.062)	.034
Area (mm^2^)	294 (211-399)	1.002 (0.9996-1.004)	.12
TL/descending aorta			
By mean diameter (%)	80.3 (9.4)	0.174 (0.0001-236.5)	.63
By circumference (%)	66 (61-73)	1.001 (0.981-1.021)	.96
By area (%)	35 (26-44)	1.058 (0.143-7.842)	.96
Stent graft size diameter (mm)	26.7 (2.1)	1.319 (1.12-1.554)	.0009
Stent graft length		1.035 (0.988-1.085)	.15
60 mm	11 (13.1%)		
90 mm	11 (13.1%)		
120 mm	62 (73.8%)		
Level of distal landing zone of FET		1.373 (0.633-2.976)	.42
Th5	11 (13.1%)		
Th6	24 (28.6%)		
Th7	49 (58.3%)		
Stent graft/mean AD (%)	85.7 (7.4)	0.035 (0.00001-155)	.43
Stent graft/maximum TL diameter (%)	104 (100-114)	1.001 (0.955-1.049)	.97
Operative time (min)	339 (284-432)	0.999 (0.994-1.003)	.53
CPB time (min)	220 (182-269)	0.996 (0.988-1.004)	.38
Cardiac arrest time (min)	134 (117-168)	0.997 (0.989-1.005)	.48
Circulatory arrest time (min)	39 (30-50)	1.018 (0.989-1.048)	.22

Abbreviations: AD, aortic diameter; CI, confidence interval; CPB, cardiopulmonary bypass; d-SINE, distal stent graft-induced new entry; sub-HR, subdistribution hazard ratio; TL, true lumen.

**Figure 2.**
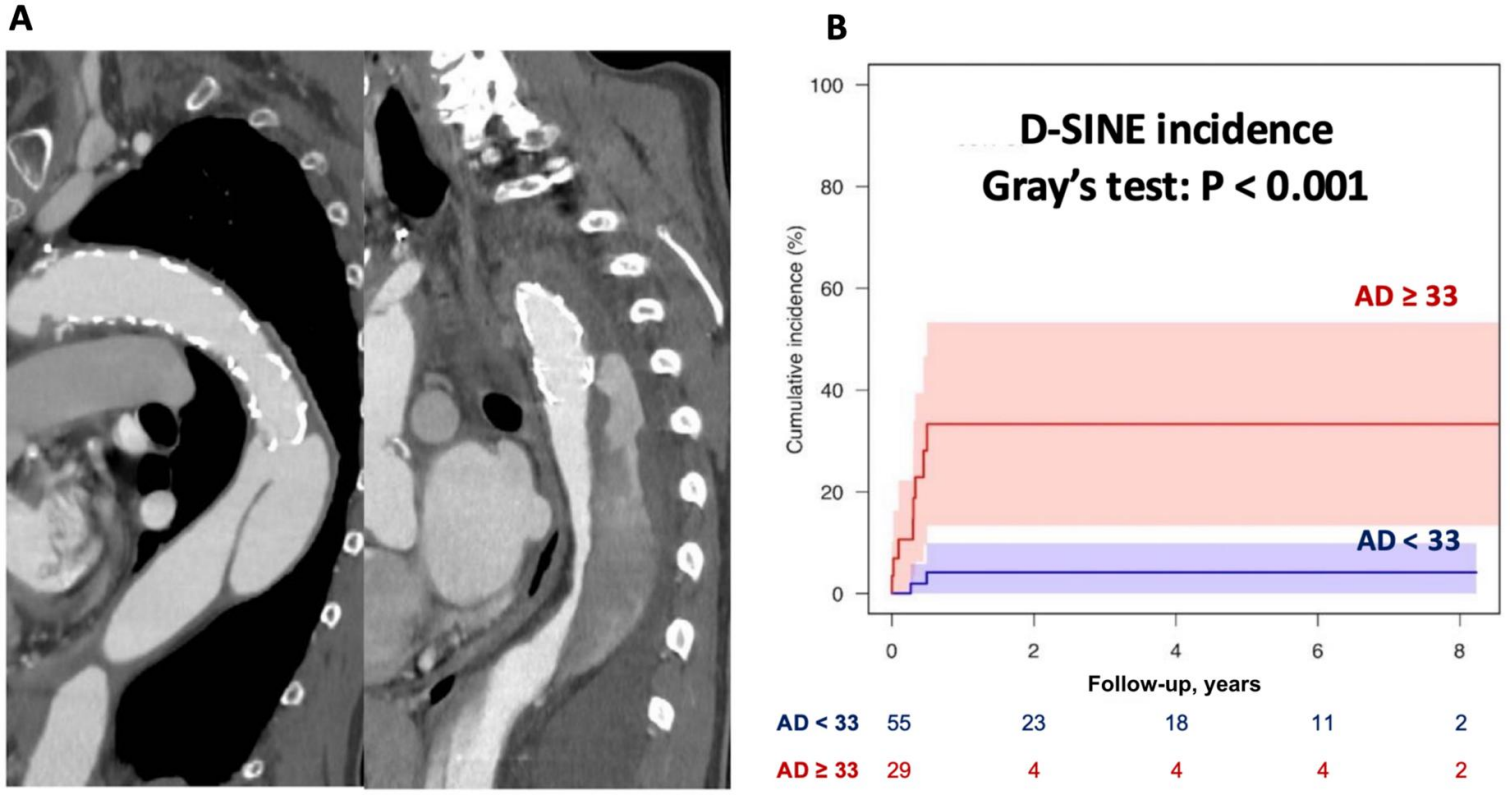
(A) Typical CT images of d-SINE following FET procedure. (B) Competing risk regression analysis for d-SINE occurrence, comparing descending aortic diameter <33 mm and ≥33 mm, with death as a competing event. d-SINE, distal stent graft-induced new entry; FET, frozen elephant trunk

**Figure 2A** demonstrates representative CT images of a patient diagnosed with d-SINE. Patients with a descending aortic diameter ≥33 mm had a significantly higher incidence of d-SINE (Gray’s test, *P* < .001). Fine and Gray analysis confirmed this association (sub-HR, 8.629; 95% CI; 1.866-39.91; *P* = .0058), as demonstrated in **Figure 2B**.

**Figure 3.**
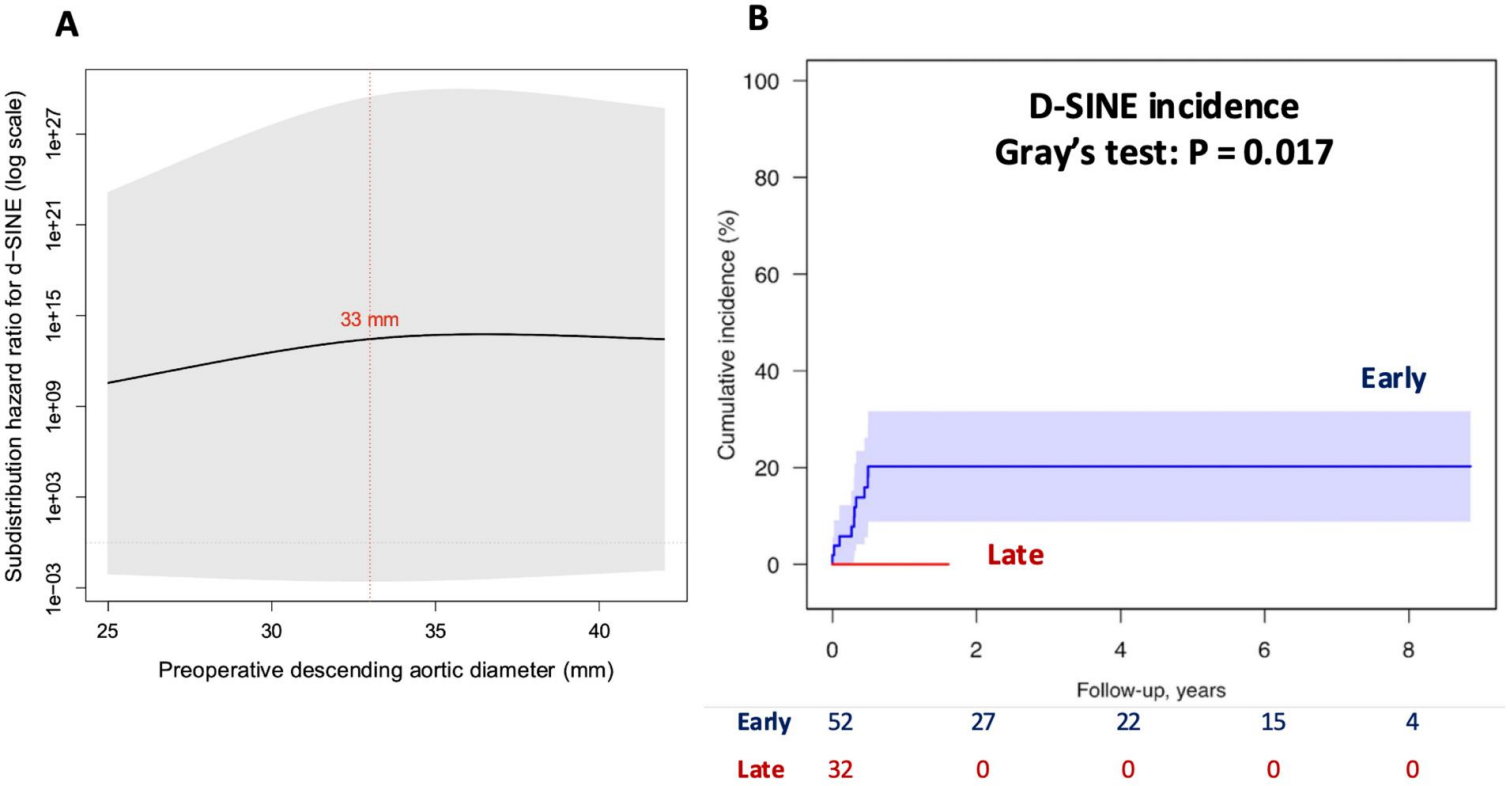
(A) Restricted cubic spline curve illustrating the nonlinear association between preoperative descending aortic diameter and the sub-HR for d-SINE occurrence. The curve was derived from fine and gray competing risk regression incorporating a restricted cubic spline with 2 knots. The sub-HR is plotted on a logarithmic scale. The red dashed line indicates the 33 mm threshold. Shaded areas represent 95% confidence intervals. d-SINE, distal stent graft-induced new entry; sub-HR, subdistribution hazard ratio. (B) Competing risk regression analysis for d-SINE occurrence, comparing the early and late eras, with death as a competing event. d-SINE, distal stent graft-induced new entry

To further explore the potential nonlinear association between aortic diameter and d-SINE risk, a restricted cubic spline model was incorporated into the regression analysis. The spline curve showed a gradual increase in sub-HR at higher diameter values, suggesting a possible nonlinear trend. However, this trend did not reach statistical significance (*P* = .10), and the 95% CIs widened substantially in the upper range (eg, 0.82-8.41), reflecting limited event counts and statistical uncertainty. The full curve is shown in **Figure 3A**.

### Operative and postoperative data

#### Surgical factors

Descriptive values of surgical variables are summarized in **Table 3**. The implanted FET diameter was descriptively larger among patients who developed d-SINE (median: 28.6 mm) compared to those who did not (median: 26.5 mm); however, these figures are presented for descriptive reference only, without formal statistical comparison, to avoid time-dependent bias. Fine and Gray competing risk regression was used to assess the impact of surgical sizing parameters, but neither the degree of size mismatch between the SG and the descending aortic diameter (*P* = .43), nor that with the TL length (*P* = .97), showed significant association with d-SINE development. In the late era, smaller FET diameters were more frequently selected (*P* = .007), and PET usage increased significantly (*P* < .001), as shown in **Table 3**.

**Table 3.** Comparison of Baseline Characteristics, Preoperative CT Parameters, and Operative Data between the Early and Late Eras

Characteristic	Early (*N* = 52)	Late (*N* = 32)	*P*-value
Age (years)	61.3 (12.6)	62.3 (10.9)	.71
Male	37 (71.2%)	19 (59.4%)	.34
BSA (m^2^)	1.78 (1.63-1.91)	1.78 (1.54-1.90)	.32
Condition of the false lumen			.08
Open	28 (53.9%)	14 (43.7%)	
Partially thrombosed	22 (42.3%)	12 (37.5%)	
Completely thrombosed	2 (3.8%)	6 (18.8%)	
Size of the descending aorta			
Diameter (mm)	31.3 (4.5)	31.6 (2.7)	.67
Size of the TL			
Maximum diameter (mm)	25.0 (22-28)	24.5 (22.3-27)	.61
Circumference (mm)	70 (60-77)	68 (62-77)	.73
Stent graft size diameter (mm)	27.2 (2.3)	26.0 (1.4)	.007
Stent graft/mean AD (%)	87.4 (7.4)	82.8 (6.3)	.004
Stent graft/maximum TL diameter (%)	107 (100-115)	104 (100-114)	.27
Using of PET	0	11 (34.4%)	<.001
1-year d-SINE occurrence (%)	20.3	0	.009
3-year d-SINE occurrence (%)	20.3	–	
5-year d-SINE occurrence (%)	20.3	–	
1-year all-cause mortality (%)	3.9	0	.27
3-year all-cause mortality (%)	3.9	–	
5-year all-cause mortality (%)	3.9	–	

Abbreviations: AD, aortic diameter; BSA, body surface area; d-SINE, distal stent graft-induced new entry; PET, J Graft Frozenix Partial ET; SD, standard deviation; TL, true lumen.

#### Follow-up outcomes

The mean follow-up duration was 2.8 years (median 1.3 years, maximum 8.9 years). The median follow-up period was 4.2 years (IQR 1.2-6.4) for patients operated on in the early era, and 0.9 years (IQR 0.6-1.4) for those in the late era. However, postoperative CT beyond 1 year could not be performed in 18 patients (21.4%) due to loss to follow-up or clinical limitations. Among the 10 d-SINE cases, 2 occurred early postoperatively. Eight underwent TEVAR, and none required open graft replacement. Distal FL control was confirmed by CT in all but the 2 early cases. No cases of aortic rupture or rupture-related mortality were observed during the follow-up period. One patient developed paraparesis after the initial surgery; however, no additional cases of paraplegia or new paraparesis were reported after TEVAR. Furthermore, 1 patient developed a SG infection after TEVAR and subsequently required open surgical removal of the infected graft. In patients without additional TEVAR, aortic remodelling at the distal FET site was assessed between the immediate postoperative period and 1-year follow-up. As shown in **Figure S2**, the descending aorta decreased in diameter (31.8 mm [SD 3.5] to 28.9 mm [SD 2.8], *P* < .001), while the SG expanded (25.3 mm [SD 2.6] to 27.1 mm [SD 2.1], *P* < .001). As shown in **Figure 3B**, the d-SINE incidence was significantly lower in the late era (*P* = .017).

#### Predictive parameters of d-SINE

After excluding variables with high multicollinearity, Fine and Gray competing risk regression analysis identified a preoperative descending aortic diameter ≥33 mm as an independent predictor of d-SINE (sub-HR, 6.11; 95% CI, 1.176-31.740; *P* = .031) as summarized in **Table 4**. In contrast, SG size was not significantly associated with d-SINE. When stratified by preoperative descending aortic diameter, d-SINE incidence was significantly higher in patients with a diameter ≥33 mm (27.6%, 8/29) than in those with ≤32 mm (3.6%, 2/55). These findings suggest a strong association between larger preoperative aortic diameters and the development of d-SINE.

**Table 4.** Competing Risk Regression Analysis for Predictors of Distal Stent Graft-Induced New Entry

Variable	Multivariable sub-HR (95% CI)	*P*-value
Aortic diameter ≥33 (mm)	6.11 (1.176-31.740)	0.031
Stent graft size diameter (mm)	1.16 (0.9699-1.389)	0.10

Abbreviations: CI, confidence interval; sub-HR, subdistribution hazard ratio.

## DISCUSSION

This study demonstrated that the incidence of d-SINE following FET for ATAAD was 11.9%, with all cases occurring within 6 months (mean onset: 102 days [SD 69]).

The cumulative incidence at 1, 3, and 5 years remained stable at 13.7%. Notably, a preoperative descending aortic diameter ≥33 mm was identified as an independent predictor, increasing the risk by 6.11-fold.

Previous studies reported a 14% d-SINE incidence after FET for aortic dissection,^10^ consistent with our findings. However, specific anatomical predictors have remained unclear, likely due to the inclusion of both acute and chronic dissection cases.

Our results showed lower d-SINE incidence in ATAAD than in chronic dissection.^12^^,^^16^ In chronic cases, progressive fibrosis and stiffening of the FL and intimal layers limit their compliance, leading to increased mechanical stress at the distal SG edge. This structural rigidity may impair aortic remodelling and predispose patients to d-SINE. While SG oversizing is a known risk factor for TEVAR,^13–15^ it was not a significant one in this study, possibly due to the increasing use of smaller FET devices. The lower d-SINE incidence in the late era (*P* = .017) supports the role of improved device selection. Hybrid arch repair, which avoids FET, may also be a valid alternative and warrants further investigation.^20^

Unlike previous reports, this study identified a descending aortic diameter ≥33 mm as an independent predictor. Aortic diameter was analysed as a continuous variable; however, 33 mm was used descriptively as it approximated the upper quartile and aided in clinical interpretation. In addition to binary stratification at 33 mm, a restricted cubic spline model was used to explore potential nonlinear associations between descending aortic diameter and d-SINE risk. The spline curve showed a gradual increase in sub-HR at higher diameters, suggesting a possible threshold effect. However, this trend did not reach statistical significance (*P* = .10), and the wide 95% CIs in this range reflect limited events and statistical uncertainty. These findings should therefore be interpreted with caution and validated in larger studies.

Patients with aortic diameter ≥33 mm had a significantly higher d-SINE incidence (27.6% vs 3.6%), suggesting that greater radial force exerted by the SG on fragile intimal layers may contribute to its development. The fact that all d-SINE occurred within 6 months highlights this period as critical. Pre-emptive TEVAR may be justified in high-risk patients, especially those with early postoperative FL expansion, though spinal cord ischaemia remains a concern.^6^ Favourable results from staged TEVAR suggest potential, and CT surveillance every 3 months may enable timely intervention.

Low-radial-force FETs such as PET may reduce d-SINE risk. PET is designed to have a reduced radial force and spring-back force than conventional FET devices, based on manufacturer-provided data, which may contribute to a lower d-SINE incidence. In this study, PET was used in 34.4% (11/32) of the patients in the late era, with no d-SINE cases observed in this group. The use of PET may help prevent d-SINE, especially in patients with larger descending aortas, but longer follow-up is needed to confirm its consistent benefit.

At the 1-year follow-up CT, a significant reduction in the aortic diameter and a concurrent expansion of the FET diameter were observed, suggesting that a smaller FET selection may be a viable strategy. Although no significant association was found between absolute aortic diameter and d-SINE incidence, the reduction observed in the later period likely reflects a shift towards more conservative sizing, along with the increased use of lower radial force FETs and refinements in surgical technique. Postoperative SG expansion may promote favourable aortic remodelling and reduce early d-SINE risk by minimizing excessive radial force. However, concerns remain regarding the late d-SINE. Chronic aortic remodelling may cause a mismatch between the expanding SG and stiffened aortic wall, especially with thickened or thrombosed FLs, leading to delayed stress at the distal FET edge. Smaller FETs may fail to fully expand, leading to incomplete thrombosis of the FL and sustained pressurization that could trigger distal re-entry. Conversely, larger FETs may promote early TL stabilization and reduce late complications, but their higher radial force may increase stress on the aortic wall. Given these trade-offs, long-term surveillance remains crucial, particularly for smaller FETs. Further studies are needed to determine the optimal FET size selection to balance the early and late d-SINE risk while ensuring long-term aortic stability.

### Limitations

This study had a few limitations. First, as this was a single-centre, retrospective observational study, it was subject to selection bias. Second, patients who were unable to undergo follow-up CT were excluded, which may have led to an underrepresentation of factors such as connective tissue disorders that could have influenced d-SINE occurrence. Third, the exact timing of d-SINE onset remains unclear, as d-SINE is often asymptomatic and incidentally detected during routine follow-up CT or emergency imaging. Finally, temporal changes in SG sizing strategies, particularly the recent trend towards selecting smaller grafts, may have influenced both the incidence and timing of d-SINE. In addition, the shorter follow-up in this cohort may limit the interpretation of long-term outcomes.

## CONCLUSION

This study identified a preoperative descending aortic diameter ≥33 mm as an independent risk factor for d-SINE following FET for ATAAD. In high-risk patients, strategies such as pre-emptive TEVAR or the use of low-radial-force FET may help reduce the incidence of d-SINE.

## Supplementary Material

ezaf264_Supplementary_Data

## Data Availability

All data supporting the findings of this study are available within the article. No new data were generated or analysed in support of this research.

## References

[1] Yoshimura N , Sato Y, Takeuchi H, et al; Committee for Scientific Affairs, The Japanese Association for Thoracic Surgery. Thoracic and cardiovascular surgeries in Japan during 2022: annual report by the Japanese Association for Thoracic Surgery. Gen Thorac Cardiovasc Surg. 2025;73:254-294. 39937349 10.1007/s11748-024-02106-x PMC11914243

[2] Kato M , Kuratani T, Kaneko M, Kyo S, Ohnishi K. The results of total arch graft implantation with open stent-graft placement for type A aortic dissection. J Thorac Cardiovasc Surg. 2002;124:531-540. 12202870 10.1067/mtc.2002.124388

[3] Sun L , Qi R, Zhu J, Liu Y, Zheng J. Total arch replacement combined with stented elephant trunk implantation: a new “standard” therapy for type A dissection involving repair of the aortic arch? Circulation. 2011;123:971-978. 21339481 10.1161/CIRCULATIONAHA.110.015081

[4] Dohle D-S , Tsagakis K, Janosi RA, et al Aortic remodelling in aortic dissection after frozen elephant trunk. Eur J Cardiothorac Surg. 2016;49:111-117. 25715431 10.1093/ejcts/ezv045

[5] Berger T , Kreibich M, Morlock J, et al True-lumen and false-lumen diameter changes in the downstream aorta after frozen elephant trunk implantation. Eur J Cardiothorac Surg. 2018;54:375-381. 29471419 10.1093/ejcts/ezy031

[6] Kreibich M , Berger T, Rylski B, et al Aortic reinterventions after the frozen elephant trunk procedure. J Thorac Cardiovasc Surg. 2020;159:392-399.e1. 30928219 10.1016/j.jtcvs.2019.02.069

[7] Kreibich M , Berger T, Walter T, et al Downstream thoracic endovascular aortic repair following the frozen elephant trunk procedure. Cardiovasc Diagn Ther. 2022;12:272-277. 35800359 10.21037/cdt-22-99 PMC9253175

[8] Haensig M , Schmidt A, Staab H, Steiner S, Scheinert D, Branzan D. Endovascular repair of the thoracic or thoracoabdominal aorta following the frozen elephant trunk procedure. Ann Thorac Surg. 2020;109:695-701. 31470013 10.1016/j.athoracsur.2019.07.011

[9] Murana G , Costantino A, Campanini F, et al Distal stent graft-induced new entry (dSINE) after frozen elephant trunk: a scoping review. Cardiovasc Diagn Ther. 2023;13:408-417. 37583692 10.21037/cdt-22-234 PMC10423728

[10] Hiraoka A , Iida Y, Furukawa T, et al Predictive factors of distal stent graft-induced new entry after frozen elephant trunk procedure for aortic dissection. Eur J Cardiothorac Surg. 2022;62:ezac325. 35678563 10.1093/ejcts/ezac325

[11] Di Marco L , Nocera C, Snaidero S, et al Staging TEVAR after FET—an exception or the rule? Indian J Thorac Cardiovasc Surg. 2023;39:224-232. 38093927 10.1007/s12055-023-01611-7 PMC10713962

[12] Ito H , Bessho S, Shomura Y, et al Long-term results of the frozen elephant trunk technique in primary chronic type B aortic dissection. Gen Thorac Cardiovasc Surg. 2024;72:770-778. 38822182 10.1007/s11748-024-02043-9

[13] D'cruz RT , Syn N, Wee I, Choong AMTL, Singapore Vascular Surgical Collaborative (SingVaSC). Risk factors for distal stent graft-induced new entry in type B aortic dissections: systematic review and meta-analysis. J Vasc Surg. 2019;70:1682-1693.e1. 31653382 10.1016/j.jvs.2019.02.040

[14] Lortz J , Leinburger F, Tsagakis K, et al Distal stent graft-induced new entry: risk factors in acute and chronic type B aortic dissections. Eur J Vasc Endovasc Surg. 2019;58:822-830. 31628049 10.1016/j.ejvs.2019.04.015

[15] Canaud L , Gandet T, Sfeir J, Ozdemir BA, Solovei L, Alric P. Risk factors for distal stent graft-induced new entry tear after endovascular repair of thoracic aortic dissection. J Vasc Surg. 2019;69:1610-1614. 30612824 10.1016/j.jvs.2018.07.086

[16] Yamane Y , Katayama K, Furukawa T, et al Mid-term results of frozen elephant trunk technique for chronic aortic dissection. Ann Vasc Dis. 2020;13:137-143. 32595789 10.3400/avd.oa.19-00131 PMC7315230

[17] Uchida N , Katayama A, Higashiue S, et al A new device as an open stent graft for extended aortic repair: a multicentre early experience in Japan. Eur J Cardiothorac Surg. 2016;49:1270-1278. 26385983 10.1093/ejcts/ezv310

[18] Okita Y. Frozen elephant trunk with Frozenix prosthesis. Ann Cardiothorac Surg. 2020;9:152-163. 32551247 10.21037/acs.2020.03.13 PMC7298232

[19] Kanda Y. Investigation of the freely available easy-to-use software ‘EZR’ for medical statistics. Bone Marrow Transplant. 2013;48:452-458. 23208313 10.1038/bmt.2012.244 PMC3590441

[20] Hasami NA , Geuzebroek GSC, Nauta FJH, et al Staged hybrid approach for acute type A aortic dissection: zone 2 arch replacement and completion thoracic endovascular aortic repair upon indication. Eur J Cardiothorac Surg. 2025;67:ezaf081. 40073250 10.1093/ejcts/ezaf081 PMC11922548

