# Supplementary material for: Predictors of Distal Stent Graft-Induced New Entry after Frozen Elephant Trunk in Acute Type A Aortic Dissection
Source: Eur J Cardiothorac Surg. 2025 Aug 4;67(8):ezaf264. doi: 10.1093/ejcts/ezaf264 (PMC12343008; doi:10.1093/ejcts/ezaf264)
Supplement: ezaf264_Supplementary_Data [file ezaf264_supplementary_data.zip › R2 supplementary figure S2.pdf]

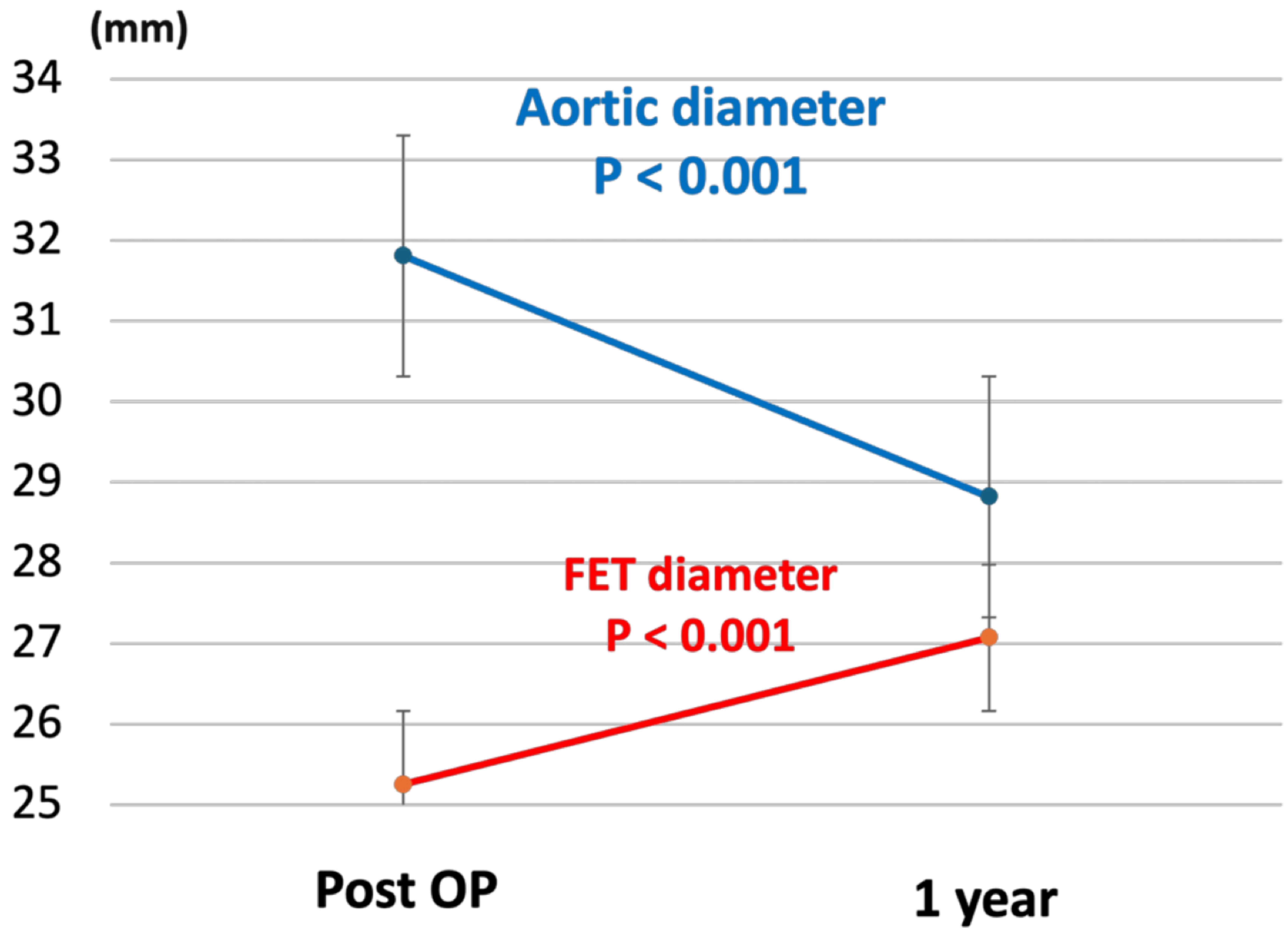

Supplementary Figure S2: Changes in descending aortic and stent-graft diameters at 1-year follow-up. The descending aortic diameter significantly decreased from 31.8 mm (SD 3.5) to 28.9 mm (SD 2.8) ($P < 0.001$), while the stent-graft diameter significantly increased from 25.3 mm (SD 2.6) to 27.1 mm (SD 2.1) ($P < 0.001$).
