# Supplementary figures and images for: Predictors of Distal Stent Graft-Induced New Entry after Frozen Elephant Trunk in Acute Type A Aortic Dissection
Source: Eur J Cardiothorac Surg. 2025 Aug 4;67(8):ezaf264. doi: 10.1093/ejcts/ezaf264 (PMC12343008; doi:10.1093/ejcts/ezaf264)

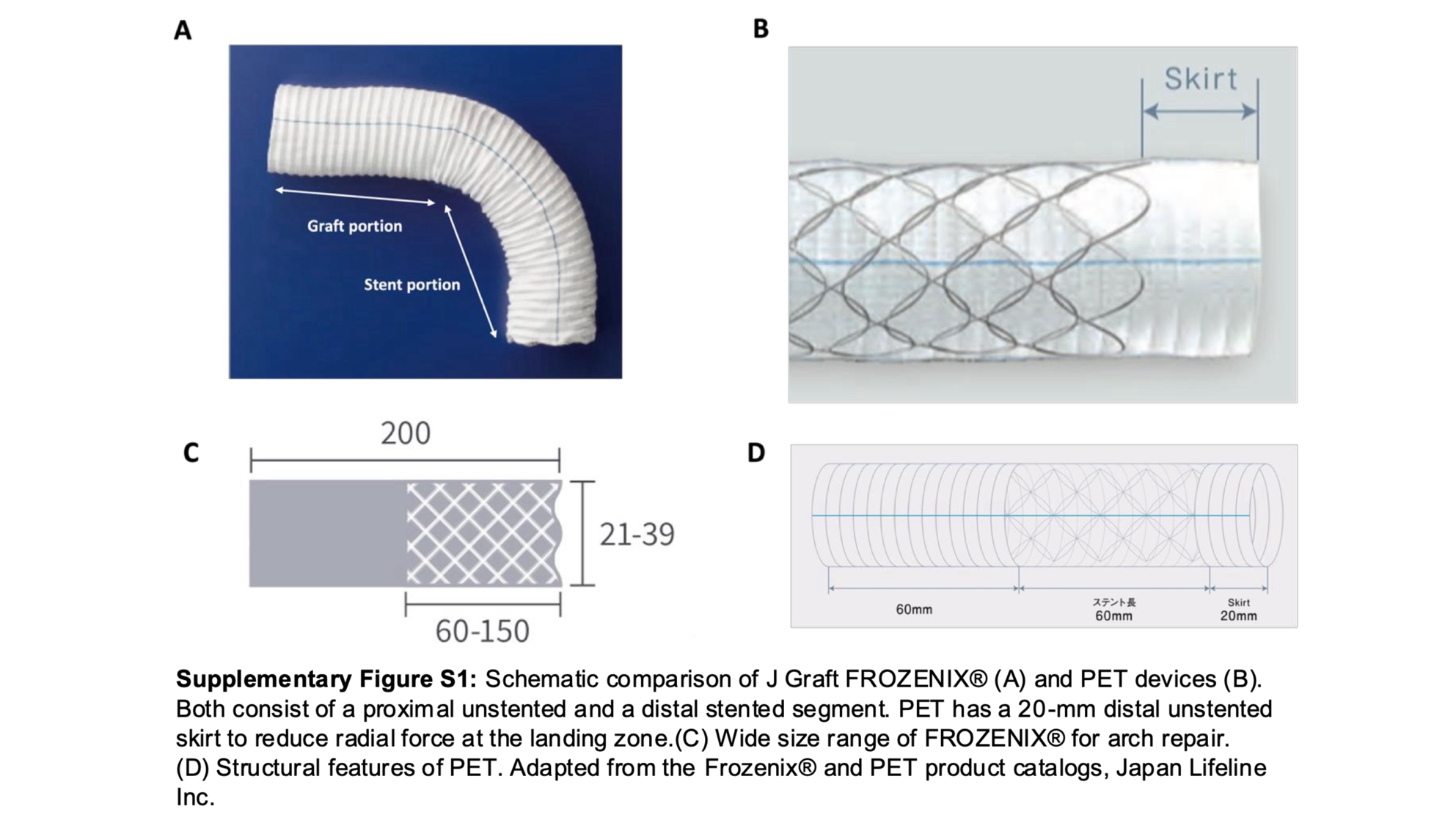

Supplement: ezaf264_Supplementary_Data [file ezaf264_supplementary_data.zip › Replaced supplementary figure S1.png]
